# Abundance of Transgene Transcript Variants Associated with Somatically Active Transgenic *Helitrons* from Multiple T-DNA Integration Sites in Maize

**DOI:** 10.3390/ijms24076574

**Published:** 2023-03-31

**Authors:** Chuxi Li, Chunsheng Cong, Fangyuan Liu, Qian Yu, Yuan Zhan, Li Zhu, Yubin Li

**Affiliations:** 1 Biotechnology Research Institute, Chinese Academy of Agricultural Sciences, Beijing 100081, China; lichux@126.com (C.L.); congchunsheng@126.com (C.C.); zhuli01@caas.cn (L.Z.); 2 College of Agronomy, Qingdao Agricultural University, Qingdao 266109, China; 20202201014@stu.qau.edu.cn (F.L.); yuqian@qau.edu.cn (Q.Y.); zy11296224@163.com (Y.Z.)

**Keywords:** maize, *Helitron*, transcript variation, somatic excision, circular intermediate

## Abstract

*Helitrons*, a novel type of mysterious DNA transposons discovered computationally prior to bench work confirmation, are components ubiquitous in most sequenced genomes of various eukaryotes, including plants, animals, and fungi. There is a paucity of empirical evidence to elucidate the mechanism of *Helitrons* transposition in plants. Here, by constructing several artificial defective *Helitron* (*dHel*) reporter systems, we aim to identify the autonomous *Helitrons* (*aHel*) in maize genetically and to demonstrate the transposition and repair mechanisms of *Helitrons* upon the *dHel-GFP* excision in maize. When crossing with various inbred lines, several transgenic lines produced progeny of segregated, purple-blotched kernels, resulting from a leaky expression of the *C1* gene driven by the *dHel*-interrupted promoter. Transcription analysis indicated that the insertion of different *dHels* into the *C1* promoter or exon would lead to multiple distinct mRNA transcripts corresponding to transgenes in the host genome. Simple excision products and circular intermediates of *dHel-GFP* transposition have been detected from the leaf tissue of the seedlings in F_1_ hybrids of transgenic lines with corresponding *c1* tester, although they failed to be detected in all primary transgenic lines. These results revealed the transposition and repair mechanism of *Helitrons* in maize. It is strongly suggested that this reporter system can detect the genetic activity of autonomic *Helitron* at the molecular level. Sequence features of *dHel* itself, together with the flanking regions, impact the excision activity of *dHel* and the regulation of the *dHel* on the transcription level of the host gene.

## 1. Introduction

*Helitrons* are DNA transposons that were first discovered by the computational analysis of eukaryote genomes and shown to be ubiquitous in fungi, plants, and animals with later experimental confirmations [1,2,3,4,5,6]. *Helitrons* lack the typical structural features of classic DNA transposons, such as terminal inverted repeats (TIRs) or target site duplications (TSDs) upon insertion, making it more challenging for precise annotation. *Helitrons* possess few constant structural and sequence features instead, including conserved 5′-TC and 3′-CTRR termini as well as palindromic sequences near the 3′ terminus. *Helitrons* insert mainly into the AT host dinucleotides. Furthermore, the continuous, incremental improvements of algorithms have allowed the identification of an increasing number of *Helitrons* from unceasing genomic sequencing efforts [7,8,9,10]. In addition, bioinformatic analysis established that *Helitrons* also exhibit sequence conservation over a 30-bp stretch from either their 5′ or 3′ terminus, which is oftentimes one of the major criteria for grouping novel *Helitron* elements [11,12,13,14].

The variation caused by *Helitron* activity in plants mainly consists of insertional mutations and consequent haplotype diversity from colinearity violation of genic *Helitron* transposons. *Helitron* transposons tend to insert themselves into the regulatory regions of genes, such as in promoters [15,16,17], introns [7,18], and 3′ untranslated regions (3′ UTRs) [19], which often leads to a loss of function in the affected gene and changes in traits. For instance, a 6.5-kb *Helitron* element was identified in the proximal promoter region of *barren stalk1* (*ba1-ref*), leading to defective developments of axillary meristems in maize [15]. The insertion of *Helitrons* can also create new functions and traits in the host, which is an important driving force of genome variability and evolution [20].

The identification of structurally similar *Helitron* transposons in a growing number of genomes [18,21] lends further support to the hypothesis that *aHel* elements are genetically active in diverse genomes [10,22,23,24,25,26]. In plants, *Helitron* transposons appear to be exclusively non-autonomous and sometimes carry other gene fragments with protein-coding potential outside of the open reading frame encoding a defective RepHel (replication initiation protein and helicase domain) transposase [8,9,15,17,27].

Due to their lack of significant structural and sequence features compared to other transposable elements, it is extremely challenging that the identification of *Helitron* transposons in a given genome relies heavily on thorough mining and annotation of the genome. The only plausible conserved sequences at both termini and 3′-end stem-loop structure are the main basis for the identification of *Helitrons* [11,12,13,28,29,30,31,32]. Notably, the development of high-throughput sequencing technologies and a better understanding of *Helitron* structure have facilitated the discovery of many non-autonomous *Helitron* transposons and a few suspected examples of *aHel* in plant and animal genomes [1,2,3,5,13,26]. Recently, Grabundzija et al. have successfully assembled an autonomous *Helitron* transposon, named *Helraiser*, from *Myotis lucifugus* [10,33], demonstrating transposon activities in vitro via the previously proposed transposition mechanism of rolling-circle replication (RCR). Furthermore, Kosek et al. reported the cryo-electron microscopy structure of the *Helraiser* transposase and demonstrated the tightly packed assembly formed by monomeric *Helitron* transposase to bury the covalently attached cleaved end and protect it until the second end becomes available [34].

Nevertheless, in maize (*Zea mays* L.), Li and Dooner [35] detected the unexpected somatic excision of various non-autonomous *Helitrons* from multiple genetic loci differed in inbred lines. Among them, the 6-kb *HelA2 Helitron* transposon was excised, leaving footprints of a variable number of TA repeats at the original insertion site. These results suggested that *Helitrons* may also transpose through a cut-and-paste mechanism in addition to the RCR transposition mechanism. *Helitrons* are widespread DNA transposons in eukaryotes; however, their distribution was significantly different among organisms. For instance, the total number and classifications of *Helitron* transposons in rice (*Oryza sativa*) differ from maize genomes, as revealed by bioinformatic analysis with the HelitronScanner [13]. There are hundreds of tandemly arrayed and truncated *CentHel* elements in rice; however, the number of *CentHel* in the same category in maize was fewer than six. Thereby, the cut-and-paste transposition mechanism [35] may be more prevalent in maize than in rice [36]. While the molecular structure of autonomous *Helitrons* and their replicative transposition via rolling-circle replication mechanism—particularly in animals—has been intensively studied, further investigations are much need to verify the prevalence of the cut-and-paste transposition mechanism and genetically define and molecularly clone the autonomous *Helitrons,* practically in plants.

Here, we constructed several maize *Helitron* systems to demonstrate their transposition activity of the cut-and-paste mechanism, excision footprint repairing mechanism, and sequence preferences upon reinsertion of excised *Helitrons*. In this artificial system, green fluorescent protein (*GFP*) is driven by the promoter of an endosperm-specific maize gene and flanked by both 5′-end and 3′-end conserved terminal sequences from either high-copy-number or genetically active non-autonomous *Helitrons* in maize. To phenotypically monitor the transposition activity of *aHel* in maize, the *dHel-GFP* elements are placed in a gene cassette of *Colored aleurone 1* (*C1*), an indispensable transcription factor responsible for pigmentation in aleurone of maize kernels. We transformed the construct into a *c1* donor inbred line via *Agrobacterium tumefaciens*–mediated transformation, identified GFP-positive transformants and crossed to other *c1* maize inbred lines or hybrids from different inbred lines. The somatic excision of *dHel-GFP* transposons was monitored by the occurrence of the segregation of purple-spotted kernels resulting from the presence of *aHel* and the existence of a cut-and-paste transposition mechanism. These genetically identified *aHel* could then be cloned in the corresponding segregating populations for further characterization of their sequence structures, the genetic basis of either the rolling-circle-replication or cut-and-paste transposition mechanism, their repair mechanism upon excision, and their sequence preference for new insertion sites. Tackling these questions will lead to a greater understanding of *Helitron* biology and its application in genome engineering. Furthermore, the cloning of autonomous *Helitrons* in maize and the generation of efficient mutant collection from *dHel-GFP* insertions will spur the development of reverse genetic resources in maize functional genomic studies.

## 2. Results

### 2.1. Phenotypic Variation Correlated with Transgene Expression in Primary Transformants and Their Hybrids with c1 Tester Lines

**Figure 1:**
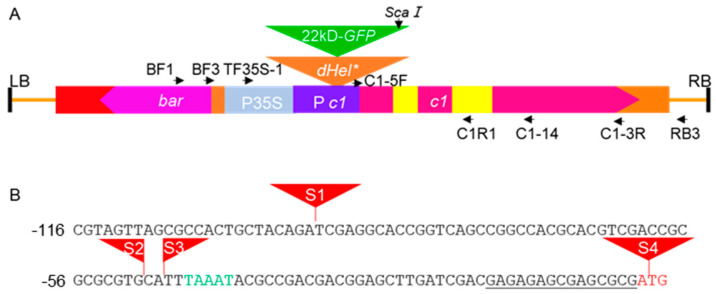
Schematic representation of the T-DNA construct used in Agrobacterium transformation. (**A**) Schematic diagram of the *c1-dHel-GFP* T-DNA construct used in *Agrobacterium*-mediated transformation of Hi II (*c1*) or B104 (*c1*) embryos. LB, left border; RB, right border. Horizontal arrows, primers for PCR and RT-PCR; vertical arrow, *ScaI* restriction site. (**B**) Sequence features of *dHel-GFP* insertion sites in the *c1* gene. The start codon is in red. The 5′ UTR is underlined. The TATA box is in green. The positions of the *dHel-GFP* inserts tested are shown as red triangles numbered from S1 to S4. The S2 site is a GC dinucleotide, whereas all other sites are typical AT dinucleotides.

**Figure 2:**
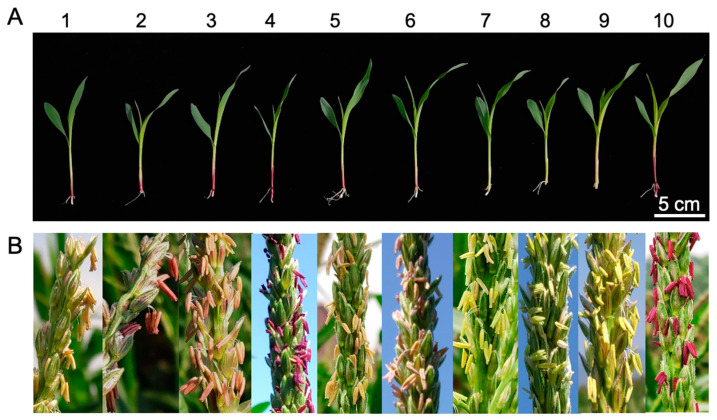
T_2_ transgenic seedlings and their anther phenotypes. (**A**) Representative T_2_ seedling phenotypes. (**B**) Representative anthers of T_2_ transgenic plants. 1, S3-Hel1613; 2, S3-Hel1158, 3, S3-HelA2; 4, S1-Hel1-4; 5, S2-Hel1-4; 6, S3-Hel1-4; 7, S4-Hel1-4; 8, B104 donor line; 9, Hi Ⅱ donor line; 10, positive *C1* transgenic line.

We created several DNA constructs to answer long-standing questions regarding autonomous *Helitron* transposons in maize by combining genetic analysis and transgenic approaches to detect the genetic behaviors of transformed defective *Helitrons* in various genetic backgrounds upon crossing with numerous inbred lines (Figure 1). Surprisingly, we found that the majority of T_2_ *c1*-*dHel-GFP* transgenic seedlings show various degrees of purple pigmentation on their coleoptiles (Figure 2(A1–A6), Table 1, Table S1), in contrast to the colorless coleoptiles of seedlings of either donor lines or non-transgenic segregants, except for the transgenic lines from construct S4-Hel1-4, of which *dHel* insertion site is right at the translation start site of *C1* gene (Figure 1B, Figure 2(A7)). Meanwhile, T_2_ *c1*-*dHel-GFP* transgenic anthers show a varied intensity of purple pigmentation upon anther emergence except for the transgenic lines from construct S4-Hel1-4 which represents its donor line of B104, in line with the pigmentation patterns on the coleoptiles (Figure 2B), regardless of the shared colorless kernel phenotype in all T_2_ ears (Figure S1).

**Figure 3:**
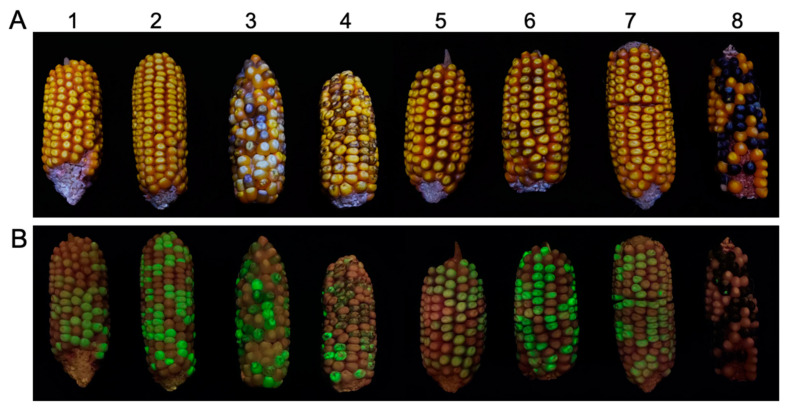
Kernel phenotype of test-cross ears. (**A**) Representative kernel pigmentation phenotypes of test-cross ears of the *c1* tester line with primary transformants from various constructs. Anthocyanin pigmentation is varied in test-cross ears from primary transformants harboring individual *c1-dHel-GFP* constructs crossed to the *c1* tester. (**B**) Representative kernel phenotype of GFP fluorescence segregation from the same test-cross ears as in (**A**) detecting under blue light illumination. 1, S3-Hel1613; 2, S3-Hel1158; 3, S3-HelA2; 4, S1-Hel1-4; 5, S2-Hel1-4; 6, S3-Hel1-4; 7, S4-Hel1-4; 8, positive *C1* transgenic line.

When crossing the T_2_ transgenic lines to the *c1* tester line, the F_1_ progenies possess interesting kernel phenotypes. For instance, all F_1_ kernels derived from crosses between the *c1* tester and transgenic plants harboring the S3-Hel1613 transgene (F_1_-S3-Hel1613 thereafter) or F_1_-S4-Hel1-4 are colorless, regardless of if they are GFP segregants. On the other hand, we detected pale purple seeds, which also showed green fluorescence, in the F_1_ test ears of F_1_-S1-Hel1-4, F_1_-S2-Hel1-4, F_1_-S3-Hel1-4, F_1_-S3-Hel1158, and F_1_-S3-HelA2 (Figure 3).

Additionally, in the BC_1_F_1_ populations for assessing the presence of autonomous *Helitrons* in tested inbred lines (Table S2), purple kernels are being segregated from crosses with inbred lines of Ky228, HP72-11, 4722, Va102, Va35, Chang7-2, CML 277, A554, P39, IA5125, and C13, respectively. However, the purple kernel phenotype is independent of green fluorescence, suggesting the presence of a wild-type *C1* gene in these inbred lines, which makes it unlikely precise excision in the transgenic *c1*-*dHel-GFP* constructs. Moreover, unilateral cross incompatibility (UCI) occurs between the BC_1_ generation (as pollen donor) and the inbred lines of CML 220, CML 321, HP72-11, and HP301, an expected result elucidated in recent publications [37]. However, no purple-spotted kernels are found on the BC_1_F_1_ segregants when all four *Ga1-S* inbred lines crossed with transgenic BC_1_ plants.

In the panel of tested inbred lines, CML 11, CML 328, and CML 331 are known for carrying *C1-I* allele [38], when crossing with W22, BC_1_-S1-Hel1-4, BC_1_-S2-Hel1-4, BC_1_-S3-Hel1-4, BC_1_-S4-Hel1-4, BC_1_-S3-Hel1613, BC_1_-S3-Hel1158, and BC_1_-S3-HelA2, all BC_1_F_1_ hybrid seeds are colorless as expected. When crossing with other inbred lines in our testing panel, the kernel pigmentation phenotype of the resulting BC_1_F_1_ segregants was similar to the kernel phenotype in F_1_ populations (T_2_ transgenic line × *c1* tester) as exampled in Figure 3. Both BC_1_F_1_-S3-Hel1613 and BC_1_F_1_-S4-Hel1-4 progenies are colorless kernels without purple spots, regardless of the green, fluorescent kernel phenotype segregation. However, green, fluorescent BC_1_F_1_ progenies of transformants from the other five constructs are all purple-mottled phenotypes.

### 2.2. Transcript Spectrum of c1 Transgenes from Variable dHel-GFP Insertion Sites

The unexpected pigmentation phenotypes in the coleoptile and anthers of T_2_ transformants from several *c1*-*dHel*-*GFP* constructs (Figure 2) or among the F_1_ kernels of T_2_ × *c1* tester and BC_1_F_1_ kernel segregants (Figure 3, Table 1) ushered in scrutinizing the *C1* transcripts from *c1*-*dHel*-*GFP* transgenes via RT-PCR and rapid amplification of cDNA ends (RACE) with total RNA extracted from T_2_ transgenic seedlings of all seven constructs (Table S3). RT-PCR assays showed an unpredicted expression of the *c1*-*dHel-GFP* transgene in T_2_ transformants from all seven constructs as the positive *C1* transgene control and the predictable absence of the *c1* expression in the negative controls of B104 and HiII donor lines (Figure S2). Notably, RT-PCR products from the *S4-Hel1-4* construct transgene are much longer than those from all other six constructs. Further sequence analysis of the cloned RT-PCR products was in line with the purple pigmentation phenotype in both coleoptile and anthers of the corresponding transgenic lines. 

**Figure 4:**
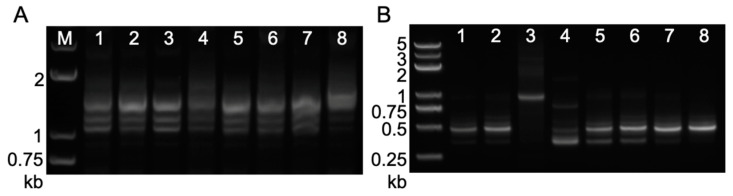
Results of RACE for *C1* transcripts from T_2_ transgenic leaf tissue. (**A**) 3′ RACE and (**B**) 5′RACE results for the *C1* transcript from total RNA extracted from leaf tissue of T_2_ transgenic plants. 1, S3-Hel1613; 2, S3-Hel1158; 3, S3-HelA2; 4, S4-Hel1-4; 5, S2-Hel1-4; 6, S3-Hel1-4; 7, S1-Hel1-4; 8, positive *C1* transgenic line.

To elucidate the molecular bases of the difference between pigmentation phenotypes of transgenic lines from various constructs, we constructed *C1* cDNA using both the 3′ RACE and 5′ RACE protocols. The generation of *C1* cDNA from the positive *C1* transgene control by the RACE protocols confirms the presence of *C1* transcripts in the tested tissue from the transgenic plants (Figure 4). Nucleotide sequence analysis of the *C1* cDNA has defined the 5′ end and the 3′ end of each of the clones. All sequenced clones of the 5′ end from the positive *C1* transgene control were identical to the endogenous *C1* cds (AF320613 and AF320614) in terms of the transcription start site and the intron-exon splicing sites (Figure 3B and Figure 4A). At the 3′ end, we identified two major transcripts resulting from alternative polyadenylation differed by 54 bp (Figure 4A and Figure S3), 618 bp and 564 bp downstream of the stop codon, respectively (Figure S4).

Three types of shorter 3′ UTR sequences were detected across the seven *c1*-*dHel-GFP* transgenic lines (Figure 4A and Figure S3). Sequence analysis showed that they mainly differed from the positive *C1* transgene control or one another at the alterable polyadenylation sites (Table S4). The majority of polyadenylation sites of the clones generated by the RACE protocol spanned an area of 400 nucleotides annotated as an *hAT* element (Figure S4). Nevertheless, the detected 3′-end coding regions were intact from all transgenic lines, suggesting that none of the *dHel-GFP* insertions affected the transcription of the 3′-end of the transgene. 

**Figure 5:**
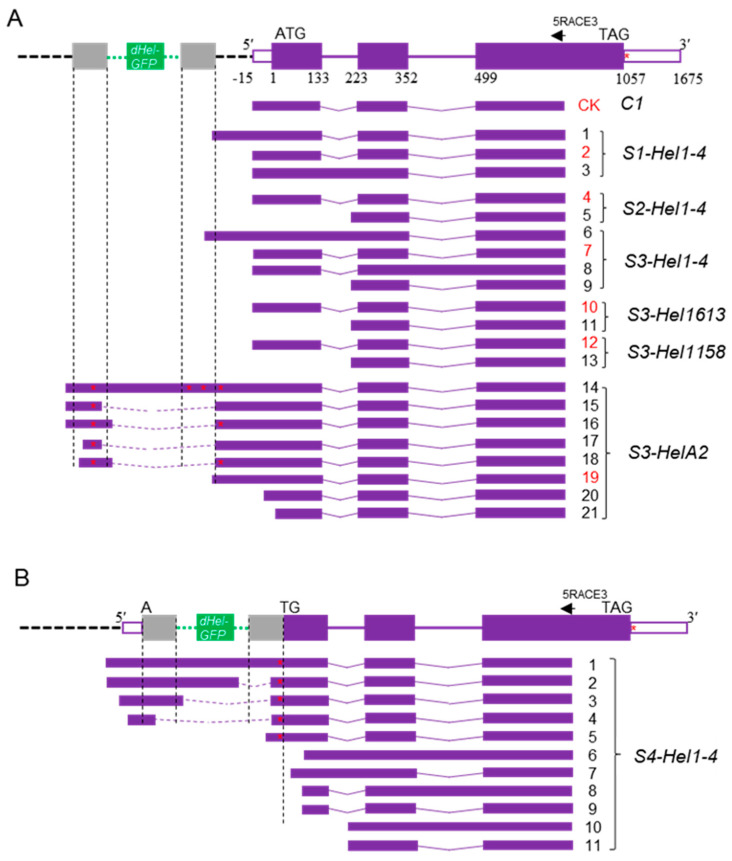
Schematic representation of *C1* transcript variants detected by 5′ RACE from T_2_ transgenic leaf tissue. (**A**) *c1* transcripts detected in total RNA extracted from leaf tissue of T_2_ transgenic plants harboring *dHel* insertions in the *c1* promoter region (sites S1 to S3), putative wild type *C1* transcripts are labeled with red numbers; (**B**) *c1* transcripts detected in total RNA extracted from leaf tissue of T_2_ transgenic plants harboring the *dHel* insertion at the translation start site of the *c1* gene (site S4). *GFP* promoter (*α-zein* promoter) and terminator (CAMV 35S terminator) are indicated by green dotted lines. LTS and RTS of *dHel* are indicated by dark vertical dashed lines. *C1* exons and introns are indicated by purple boxes and lines respectively. The corresponding intron-exon junction sites are labeled with numbers beneath. *C1* promoter region is indicated by black dotted line. 5′ and 3′ UTR are represented by open boxes. Alternative splicing events at non-canonical sites are shown with broken lines, whereas splicing events at canonical sites are shown with solid lines. *dHels* are indicated by grey boxes, and *GFP* is indicated by green boxes. Horizontal arrows, primer for 5′ RACE. Red asterisks, stop codons.

In contrast to the multiple 3′ RACE products resulting from alternative polyadenylation, the 5′ RACE products detected in the transgenic lines were greatly influenced by *dHel-GFP* insertions at either the promoter region or the translation starting site (Figure 1B, Table 1 and Figure 5B). The 5′ RACE sequence analysis confirms that the 5′ UTR of transgenic lines from constructs of S2-Hel1-4, S3-Hel1-4, S3-Hel1158 and S3-Hel1613 is 15 bp in length (Figure 5A, transcript variants No.4, 7, 10 and 12, Table S5), identical to the positive *C1* transgene control (Figure 5A CK) and the canonical 5′ UTR of the endogenous *C1* transcript. Additionally, other uncanonical variants of transcripts are readily detected, including possible novel upstream promoter (Figure 5A, transcript variant No.6), intron retention (Figure 5A, transcript variants No.6 and 8), additional exonic sequences (Figure 5A, transcript variants No.5, 9, 11, and 13, Table S5) due to alternative splicing, and unlikely to be translated to functional proteins.

The canonical 5′ UTR is also identified from transcripts of S1-Hel1-4 transgenic lines (Figure 5A, transcript variant No.2, Table S5), in addition to transcripts characterized with retained intron 1 (Figure 5A, transcript variant No.3) or transcripts initiated possibly in the 3′ end of *Hel1-4* and transcribed at 16-bp upstream of the insertion site without altering both introns-exon splicing sites in the downstream region (Figure 5A, transcript variant No.1, Table S5).

Several novel transcripts of splicing variants were cloned with the amplicon mixture from S3-HelA2 transgenic line sharing the same site (AT di-nucleotides) as in the S3-Hel1613, S3-Hel1158, and S3-Hel1-4 lines. The major amplicon was a mixture of multiple splicing variants with similar lengths but different sequence compositions from the S3-HelA2 transformant (Figure 4(B3), Table S5). The eight variants of sequenced transcripts, they share the same downstream intron-exon junctions in the *c1* coding region as the wild type *C1* gene. They mainly differed from each other by the first one or two novel exons resulting from initiating possibly in a novel promoter region (thus, in the transgenic *C1* promoter) further upstream of the 20-bp upstream of *HelA2-GFP* insertion site (Figure 5A, transcript variants No.14, 15 and 16). 

Among them, the longest transcript, although much less abundant than the major amplicons, includes the entire *HelA2-GFP* element and the downstream *c1* transcripts featured by the canonical intron-exon splicing junctions, generating a new type of chimeric transcript (Figure 5A, transcript variant No.14, Table S5). Two alternative splicing transcripts may share the same novel promoter with the longest transcript since their first exon is identical to the 5′-end of the longest transcript. However, they differ from each other with an extra intron sharing the same 3′ splice site (right at the 3′-end of *HelA2-GFP,* CTAG) but not the 5′splice sites (47 bp apart) inside the *HelA2-GFP* segment (Figure 5A, transcript variants No.15 and 16, Table S5). Two other alternative splicing transcripts, initiating right in the LTS (14 bp apart) from other possible novel promotors, share the same intron-exon splicing junctions as two previously described transcripts, respectively (Figure 5A, transcript variants No.17 and 18, Table S5). Moreover, the RTS could also be a novel promotor since one transcript initiated from 14 bp upstream of the 3′-end CTAG (Figure 5A, transcript variants No.19, Table S5). The sequences of the two shortest transcript variants detected from the S3-HelA2 transformants are different at the transcription start sites that are 3-bp apart around the ATG translation start codon, although they are much less abundant in the 5′ RACE amplicons.

The most heterogeneous transcript variants are isolated from S4-Hel1-4 transgenic lines (Figure 3B and Figure 4B), in which the *dHel-GFP* insertion site was in between an AT dinucleotide of the *C1* start codon (Figure 1B). The most abundant amplicon is the uncanonical variant of transcripts from additional exonic sequences as previously described in other transgenic lines transformed with various constructs (Figure 4, lane4; Figure 5A, transcript variants No.5, 9, 11, 13, Figure 5B transcript variant No.11 and Table S5). In addition, other ten uncanonical variants of transcripts are readily detected. For instance, the second most abundant amplicon is a transcript variant of intron retention sharing the same transcription start site as the most abundant amplicon (Figure 5B transcript variant No.10 and Table S5). The transcription start sites of four transcript variants, featured with the prevalent intron retention variations, are at three different nucleotide positions in the first exon, such as 3, 67, and 70, respectively (Figure 5B, transcript variants No.6–9, Table S5). Similar to the findings from other constructs, either the same *Hel1-4* or the different *HelA2*, the RTS of S4-Hel1-4 could also be a novel promotor since B7 transcript initiated from nucleotide position 3, evidenced with another transcript variant initiating from 67 bp upstream of the 3′-end CTAG (Figure 5B, transcript variant No.5, Table S5). All remaining four transcript variants may share the same promotor as the canonical *C1* transcript, but their transcription start sites are at a region of 12 bp to 68 bp upstream of the 5′-end TC, leading to uncanonical transcript variants of intron retention (Figure 5 B, transcript variant No.1), additional exonic sequences due to alternative splicing (Figure 5B, transcript variants No.1–4, Table S5). All detected uncanonical variants of transcripts from S4-Hel1-4 transgenic lines are unlikely to be translated to functional proteins, consistent with the colorless kernel phenotype.

A total of five of the six transgenic *c1* alleles showed relatively consistent transcript patterns in spite of their apparent difference at the insertion site of *dHel-GFP* in the promoter region (S1-Hel1-4, S2-Hel1-4, S3-Hel1-4, S3-Hel1613, and S3-Hel1158). Most transcripts were still functionally equivalent to the control *C1* transcript unless they lacked the first exon or retained part of the first intron. 

Surprisingly, the main types of transcripts seen in the transgenic lines S3-Hel1158, S3-Hel1613, and S3-Hel1-4 were the same as *C1*, whereas the S3-HelA2 transgenic line produced a variety of new transcripts encoding a protein with partial *C1* function. The above four transgenes share the same insertion site for the *dHel-GFP* cassette and have similar 5′ and 3′ end sequences, but S3-HelA2 generated completely different transcript isoforms from the other three transgenes, perhaps reflecting the *Helitron* transposon incorporated into the constructs.

The main 5′ RACE products detected from S3-HelA2 transgenic lines are quite unique compared with transgenic lines from all other constructs (Figure 4B lane 3). Two major transcripts are initiated from either 20-bp upstream of the *HelA2* insertion site or right inside the 5′-end (30-bp) of the *HelA2* element (Figure 5A, transcript variants No.15 and 18, Table S5). These two *C1* transcripts differ at their 5′ splicing sites since both 3′ splicing sites were the same and corresponded exactly to the 3′ end (AG) of *HelA2* (Figure 5A, transcript variants No.15 and 18). Other minor transcripts differ from each other with varied lengths due to various transcriptional start sites (TSSs) around the *HelA2* insertion site (Figure 5A, transcript variants No.14–21), multiple 5′ splicing sites (Figure 5A, transcript variants No.15–18), the intron retention of the whole *HelA2* element (Figure 5A, transcript variant No.14, Table S5). The majority of the *C1* transcripts in the S3-HelA2 transgenic line include the TATA box region and the *C1* promoter (Figure 5A, transcript variants No.14–19); however, the open reading frames of these transcripts had multiple translation termination codons prior to the canonical translation start site of *C1* and led to non-functional open reading frame upstream of the original *C1* open reading frame. 

Recently, the identification of evolutionarily conserved features correlated with promoter expression levels in TSSs has become an important area in understanding the control of transcription initiation and regulation in a plant, including the identification of novel patterns in the dinucleotides composing the initiator element [39]. To gain a glimpse into the alteration of general sequence patterns surrounding the TSS resulting from *Helitron* insertions, 5′ RACE assay showed that *Helitron* insertions generally did not affect the patterns in the dinucleotides composing the initiator element since highly expressed transgenes were enriched most commonly for CA, followed by CG, TG, which perfectly agrees with recent research [39]. This occurred regardless of the presence of the TATA box and the varied content of CG nucleotide between the TATA box and TSS, the initiator element (Figure 1B, Table S6).

### 2.3. Footprint Analysis on Somatic Excision of Helitrons via the Cut-And-Paste Mechanism in Transgenic Lines and Hybrids with Maize Inbred Lines 

Somatic excision of *Helitrons* has been demonstrated via PCR amplification in previous research on a previously unfavorable cut-and-paste transposition mechanism [35]. To address the possibility of genetic activity of putative autonomous *Helitrons* in maize, we utilized nested PCR to detect occasional somatic excision events from T_2_ leaves of all seven different transformants (Figure 1A, Table S3). However, we have not amplified PCR products with expected sizes of the corresponding somatic excision from the *c1-dHel-GFP* cassette in any one of the seven T_2_ transgenic lines, suggesting an absence of autonomous *Helitrons* activities from the genetic background of the donor lines (Figure S5). 

**Figure 6:**
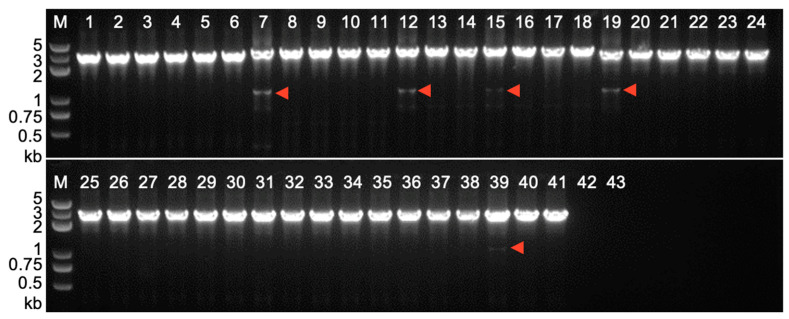
PCR amplification of somatic excision products for *dHel* in F_1_ segregants from test-crosses between different *c1-dHel-GFP* T_2_ transgenic plants and the *c1* tester line. PCR primer pairs are shown in Figure 1A. Lanes 1–6, S3-Hel1613; lanes 7–11, S3-Hel1158; lanes 12–18, S3-HelA2; lanes 19–23, S4-Hel1-4; lanes 24–28: S2-Hel1-4; lanes 29–34, S3-Hel1-4; lanes 35–41, S1-Hel1-4; lanes 42–43: F_1_ individuals from a non-transgenic line crossed to the *c1* tester. Red arrows, PCR products with the expected size for *dHel* excision products.

When the same PCR strategy was applied to DNA samples of leave tissue from various F_1_ hybrids of T_2_ transgenic lines with the *c1* tester line, we were instead able to amplify putative somatic excision products from some transgenic segregants, such as S1-Hel1-4 (Figure 6, lane 39), S3-Hel1158 (Figure 6, lane 7), S3-HelA2 (Figure 6, lanes 12 and 15), and S4-Hel1-4 (Figure 6, lane 19). Similar PCR amplicons of putative somatic excision products were also detected from several other segregants of two out of those four transgenic lines, namely S1-Hel1-4 F_1_ hybrids and S4-Hel1-4 F_1_ hybrids (Figure S6). However, we were failed to detect somatic excision products from DNA samples of leave tissue at the V2 seedling stage of more than 30 segregants of F_1_ hybrids between other T_2_ transgenic lines, S3-Hel1613, S2-Hel1-4, or S3-Hel1-4, with the *c1* tester line, respectively (Table 2). This suggests that the excision frequency of *dHel-GFP* is extremely low, in the case of S2-Hel1-4 or S3-Hel1-4, or an absence of autonomous *Helitrons* in both parental lines, in the case of S3-Hel1613.

The cloning of PCR amplicons of putative somatic excision products and the subsequent sequence analysis revealed that all somatic excisions were identical to the wild-type sequence (Table 3). The direct sequencing of the gel-purified PCR amplicons of putative somatic excision products (Figure S6) showed the same footprint as fp0. The unique excision footprint of fp0 has been reported and deemed conserved among certain *Helitron* families sharing a similar repairing mechanism upon somatic excision [12,35].

### 2.4. Circular Intermediates Detection in Rolling-Circle Transposition of Helitrons from Transgenic Lines and Their Hybrid Populations

**Figure 7:**
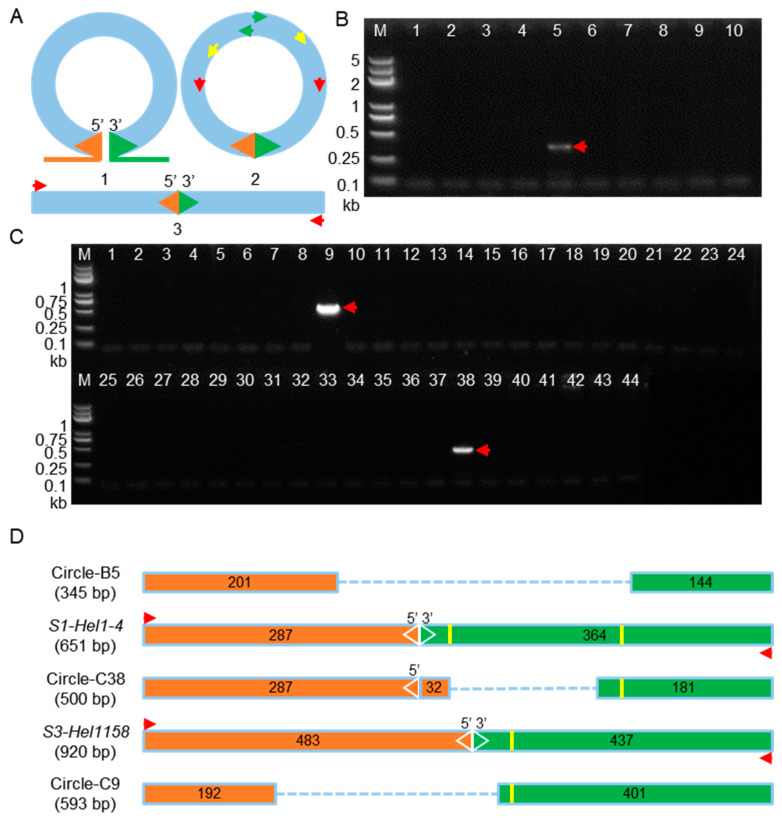
Schematic representation and PCR detection of circular *dHel* intermediates from leaf tissues of transgenic plants transformed with *c1-dHel-GFP* constructs. (**A**) Schematic diagram of circular *dHel-GFP* (1) and nested-PCR primer combinations (pairs of color-coded arrows in 2) to amplify somatic circular *dHel* intermediates (3); (**B**) Lanes 1–10, T_2_ transgenic lines from S1-Hel1-4 construct; (**C**) F_1_ segregants from a test-cross between different *c1-dHel-GFP* T_2_ transgenic plants and the *c1* tester line. Lanes 1–6, S3-Hel1613; lanes 7–11, S3-Hel1158; lanes 12–18, S3-HelA2; lanes 19–23, S4-Hel1-4; lanes 24–28, S2-Hel1-4; lanes 29–34, S3-Hel1-4; lanes 35–41, S1-Hel1-4; Lanes 42–44, F_1_ individuals from a cross between a non-transgenic line and the *c1* tester. Red arrows, PCR products of putative circular *dHel* intermediates; (**D**) Schematic representation of sequence features of circular intermediates from (**B**) and (**C**) (red arrows). The expected circular *dHel* intermediates (A3) are inverse PCR amplification of a fused 5′-end upstream sequence with 3′-end downstream sequence color-coded as orange and green, respectively. Red arrows are PCR primers, light blue dotted lines are internal deletions, vertical yellow bars are palindromic sequences upstream of the 3′-end CTAG, and the sequence configurations are labeled with numbers in the color-coded rectangles.

Recently, Grabundzija et al. constructed an active *Helitron* (“*Helraiser*”) from the genome of *Myotis lucifugus* and experimentally demonstrated the RCR transposition mechanism by sequence confirmation of the PCR amplified circular transposon intermediate containing an RTS-LTS configuration and the reinsertion of *Helraiser* in vitro [33]. To address the possibility of the existence of circular intermediates of *dHel*-*GFP* elements undergoing the RCR transposition, PCR strategy was applied to the same set of DNA samples of leaf tissue at V2 seedling stage from corresponding transgenic lines as for the somatic excision analysis (Table S3 and Figure 7A). 

We have not amplified any circular *dHel* intermediates with an expected sequence configuration of both intact LTSs and RTSs by nested PCR in either T_2_ transgenic individuals or their F_1_ hybrids with the *c1* tester line from all seven constructs (Figure S7, Table S7). Alternatively, we detected other structure derivatives of expected circular *dHel* intermediates from T_2_ individuals transformants of S1-Hel1-4 (Figure 7B lane 5) and individuals transformants from F_1_ hybrids of S1-Hel1-4 and S3-Hel1158 (Figure 7C lanes 38 and 9, respectively). 

The sequence analysis of such circular *dHel* intermediates revealed that these amplicons featured deletions at either or both ends of *dHel-GFP* elements (Figure 7D). In detail, the PCR fragment amplified in T_2_ transformant from S1-Hel1-4 is 345 bp in length, composed of 201-bp LTS and 144-bp RTS, which is much shorter than the expected length of 651-bp (287-bp LTS and 364-bp RTS), due to an internal deletion of the junction sequences of 86 bp from the LTS and 220 bp from the RTS including two stem-loop structures at the 3′-end of S1-Hel1-4 RTS (Figure 7D, Circle-B5). Furthermore, no microhomology is presented at the junction site, which is frequently found during transposition. In contrast, the circular *dHel-GFP* intermediate amplified in F_1_ individual of the same S1-Hel1-4 transformant is 500 bp in length, composed of an intact LTS and its adjacent sequence of 32 bp, and 181-bp remanent RTS due to an adjacent deletion of 183 bp proximal RTS. Notably, only one of the two stem-loop structures (TTTTTACCAAAAAA) was retained in the 181-bp remanent RTS ((Figure 7D, Circle-C38; Table S8). Moreover, we detected an imperfect microhomology (CTCAA/CTCACA) at the circularization junction between LTS and RTS. Finally, the circular *dHel* intermediate amplified from the F_1_ individual of S3-Hel1158 transformant is 593 bp in length, composed of 192-bp LTS and 401-bp RTS, which is much shorter than the expected length of 920-bp (483-bp LTS and 437-bp RTS), due to an imbalanced internal deletion of the junction sequences of 291-bp from the LTS and 35-bp from the RTS excluding the solo stem-loop structure at the 3′-end of S3-Hel1158 RTS (TAAAAAATCTTGAAATTTTTTTA, Figure 7D, Circle-C9, and Table S8). In addition, a dinucleotide microhomology (GT) was presented at the circularization junction between LTS and RTS of Circle-C9.

## 3. Discussion

### 3.1. Helitron Transposon Families and Their Insertion Sites in the Promoter Region Alter Gene Expression through Linked Processes of Transcription or Splicing

The initial objective of this research was to genetically identify the autonomous *Helitron* transposons in maize by constructing reporter systems of non-autonomous *dHel* elements to generate transgenic lines for genetic tests. We build seven *c1*-*dHel-GFP* constructs featured by four diverged *Helitron* families inserted at four dispersed sites in the promoter region of the *C1* gene to augment the possibility of identifying the genetic activities of mysterious autonomous *Helitrons* in maize (Figure 1, Table 1, Tables S1 and S2). In total, 34 transgenic events transformed with seven constructs were analyzed for phenotyping on various tissues of the individual primary transformants or their segregating populations genetically (Table 1, Table 2 and Table S1, Figure 1, Figure 2 and Figure S1), and were characterized for T-DNA integration sites, transgene copy numbers and expression pattern molecularly (Figure 3 and Figure 4, Figures S2–S4, Tables S3–S6).

The *C1* promoter has been one of the hotspots for gene function characterization in maize even in plant [40,41,42]. Different *C1* expression rates can be correlated with promoter sequence alterations in maize [43]. In current study, all four *dHel* insertion sites −93, −49, −47, and +1 relative to the start codon ATG) are located downstream of the previously identified *cis*-regulatory elements (Figure 1), such as abscisic acid (ABA) response factors and the Viviparous-1 (Vp-1) transcription factors at positions −157 to −130 relative to the transcription start site [43]. Compared to non-transgenic wild-type controls, the coleoptiles or anthers of T_2_ transgenic seedlings, segregants of the *c1* tester line crossing with transformants from constructs of S3-Hel1613, S3-Hel1158, S3-HelA2, S1-Hel1-4, S2-Hel1-4, and S3-Hel1-4, are anthocyanin pigmented, while the coleoptile of *S4-Hel1-4* T_2_ transgenic seedlings is in light green or transparent (Figure 1 and Figure 2, Table 1). Furthermore, the discrepancy between purple-pigmented coleoptile in T_2_ S3-Hel1613 seedlings and their colorless kernel phenotype indicated that the *C1* transgene expression in the aleurone layer might be blocked by the insertion of *Hel1613-GFP* but not in coleoptiles tissues, due to a possible change in the expression pattern of temporal and spatial specificity of the *C1* transgene.

We employed both RT-PCR and RACE assays to decipher the unforeseen pigmentation pattern due to possible expression of the *c1-dHel-GFP* alleles in the transformation constructs by detecting the expression pattern and cloning the full-length cDNA of the *C1* transcripts transcribed from each *dHel-GFP* transgene (Figure 3 and Figure 4, Figures S2–S4, Tables S3–S6). RT-PCR results are extremely supportive of the pigmentation pattern of different transformants, such as the colorless kernel and the light green or transparent coleoptile phenotype of the S4-Hel1-4 T_2_ transgenic seedlings. However, further RACE assays show abundant transcript variants of the S4-Hel1-4 T_2_ transgenic seedlings indicating the normal functioning of the *c1* promoter. All other three insertion sites of *Hel1-4* were free of interfering with the normal function of the *c1* promoter, which was evidenced by the similar pigmentation phenotypes and expression patterns of *C1* transgene from T_2_ transgenic segregants from transformation constructs of S3-Hel1613, S3-Hel1158, and S3-HelA2, regardless of the abundance in transcription initiation variants and splicing patterns resulting from the *dHel-GFP* insertions in the promoter region (Figure 5, Table S5). 

Combining the seed and coleoptile phenotypes, we hypothesize that *dHel-GFP* insertions into the *C1* promoter region did not completely inactivate the *C1* gene. Instead, it allowed the production of at least some canonical *C1* transcripts or 5′-truncated transcripts, as seen with the S2-HelA2 transgene that appeared to retain some *C1* function.

The BC_1_F_1_ hybrids between the inbred lines CML 11, CML 328, and CML 331 and S2-HelA2 transgenic plants also exhibited a block in anthocyanin biosynthesis in seeds. Just like the canonical *C1* transcript, the *C1* transcripts derived from the S2-HelA2 transgene also failed to induce anthocyanin biosynthesis in these BC_1_F_1_ hybrids, suggesting that the observed variation in the 5′ UTR of *C1* transcripts from S2-HelA2 does not change the inhibitory effect of upstream inhibitors on anthocyanin biosynthesis.

Based on the transcripts and phenotypes described in this study, we propose that multiple transcript variants from the host gene will appear when the insertion site of *dHel-GFP* is located in the promoter region of the host gene, even if the insertion site is conserved between transgenes, due to the differences in non-conserved sequences between different *dHels*. These variant transcripts will affect the expression of downstream target genes and ultimately lead to phenotypic differences.

The promoter of the *C1* locus or, in general, the 5′ region of the gene was under strong selection pressure, and several conserved or functional important sequence motifs have been characterized experimentally [43]. In our constructs, three *dHel-GFP* insertion sites are embedded between the insertion site of an *Enl*-induced *C1* promoter mutation *c-m668613* [44] and the TATA box for a desirably strong excision and reversion frequency without depleting the function of the *c1* gene, since previous report showed that SNPs or small indels causing depletion of the *C1* function in certain mutants [43]. However, the majority of *dHel* insertions purely affected splicing of the primary transcripts from the transgene in the transformants. Although, occasionally, the *dHel* functions as a novel promoter to drive the transcription of the transgene in the constructs, such as in the case of S3-HelA2 [45]. Furthermore, the *Helitron* insertion in different construct has no substantial effects on the patterns in the dinucleotides composing the initiator element, which is important and highly conserved to the control of transcription initiation and regulation in plant [39], but on the alternative splicing and aberrant splicing (Table S6). 

### 3.2. Coexistence of RCR/Cut-And-Paste Somatic Transposition Mechanism Catalyzed by Autonomous Helitrons in Maize

The postulated rolling circle mode of *Helitrons* transposition has been demonstrated from the recent reconstruction of *Helraiser*, an active *Helitron,* from the genome of *Myotis lucifugus*, evidenced by molecular detection and sequence confirmation of excised circular transposon intermediate that contains an RTS-LTS junction [10,33]. We have applied direct PCR and inverse PCR on transgenic segregants from 34 events transformed with seven constructs to detect excision footprints and excised circular intermediate containing RTS-LTS junctions (Figure 5 and Figure 6). First of all, PCR-amplified somatic excision products were detected from the F_1_ segregation population of T_2_ transgenic segregants with the *c1* tester line but not in T_2_ transgenic segregants (Figures S5 and S6). Among 165 transgenic segregants of 34 events transformed with seven constructs, only 12 segregants from four constructs (S1-Hel1-4, S4-Hel1-4, S3-Hel1158 and S3-HelA2) tested positive for footprint analysis (Table 2), representing three different *Helitron* families all inserted into AT dinucleotide. Sequencing analysis revealed that all footprints are fp0 (Table 3), notwithstanding the type of *Helitrons* or their insertion sites, which was not rare in endogenous *dHel* transpositions [12,35]. Nevertheless, our results support the existence of genetically active autonomous *Helitrons* and their catalytic activities to excise non-autonomous *dHel* transposons in maize. This cut-and-paste mode might be shared between somatic tissues and germ cells, although the excision frequency in either of them would be devilishly limited. 

Circularized DNA transposons, such as circularized *Ac/Ds*, circular extrachromosomal forms of *Mu*, circularized *TED* or *dTED*, and circular intermediates of *Helraiser*, are common in plants and animals [10,46,47,48]. To test whether RCR mode, which is more favorable to explain the colorless kernel phenotype in transgenic segregants such as from constructs of S4-Hel1-4 or S3-Hel1613, plays an important role in the *dHel* transposition in current *c1-dHel-GFP* systems, inverse PCR was applied to detect circular intermediates in the process of RCR transposition. Among the four constructs in which somatic excision activities have been demonstrated from the corresponding transformed segregants via footprints analysis, two of them (S1-Hel1-4 and S3-Hel1158) tested positive for amplification circular *dHel-GFP* intermediates with fused LTS and RTS in the corresponding T_2_ or F_1_ segregants. All amplified circular *dHel-GFP* intermediates are not the perfect fusion of LTS and RTS due to deletions from one or both termini featured with stretches of microhomology at the deletion junctions (Tables S7 and S8). The footprint analysis of somatic excision and the circular intermediates of *dHel-GFP* from the transgenic individuals with varied genetic backgrounds suggest the strong coexistence of rolling-circle-replication and cut-and-paste transposition mechanisms in *Helitron* transposition systems in maize.

## 4. Materials and Methods

### 4.1. Development of the Reporter System

To develop reporter systems for *Helitron* transposition activity in maize, artificial defective *Helitron* transposons labeled by *GFP* were embedded in a maize *C1* gene cassette (Figure 1A) and used for *Agrobacterium* transformation.

Four *dHel* transposons were selected to construct reporter systems for the following different reasons. The 6.0-kb *HelA2*, containing sequence fragments of three genes, was shown to excise from its original site in the chromosome *5S* of several inbred lines [27,49], leaving multifarious TA repeats as somatic excision footprints [35]. *Hel1-4* is present in the inbred lines of H99 and A188, but the somatic excision products have only been verified from H99. *Hel1158* is one of the *Cornucopious Helitrons*, the high copy-number *Helitron* families in maize [31]. *Hel1613* is a novel family of high copy-number *Helitron* other than *Cornucopious* newly identified by manual sequence annotation in maize genomes of B73 and other inbred lines.

*Hel1-4* is 469 bp in length and has been demonstrated to be capable of excising somatically in H99 [35]. In the *Hel1-4-GFP* constructs, the *Hel1-4* element was split into two parts for insertion of the *GFP* cassette, the 233-bp left terminal sequence (LTS) and the 236-bp right terminal sequence (RTS) according to the sequence conservation among elements in the same subfamily. The configuration of other *dHel-GFP* constructs is listed in Table S1.

A *GFP* expression cassette, driven by the 22-kD α zein promoter and ending with the cauliflower mosaic virus (CaMV) 35S terminator, was flanked by the LTS and RTS of each *dHel*. The *C1* coding sequence comprised a 15-bp 5′ untranslated region (UTR) and a 431-bp promoter fragment with a TATA box [42]. The individual *dHel-GFP* cassette was inserted into the *C1* promoter or exon region and led to the inactivation of the transgenic *C1* copy. Following a bioinformatics analysis, three sites upstream of the TATA box were designated as insertion sites of *dHel-GFP* (S1, S2, and S3). The fourth site, S4, was at the *C1* translation start codon (Figure 1B). The exact insertion sites of all seven constructs are given in Table 1 and Figure 1B. These constructs were transformed via *Agrobacterium* transformation method into maize inbred line B104, with an exception of S3-HelA2 into HiⅡ hybrid line.

### 4.2. Genetic Activity Detection of dHel in BC_1_F_1_ Population from Test Crosses with Inbred Lines 

The *R* and *C1* genes are indispensable in the anthocyanin biosynthesis pathway in the maize aleurone layer [50]. The lack of either the functional *C1* or *R1* gene results colorless kernels due to no anthocyanin accumulation in aleurone cells. Either transformation donor line of B104 inbred line or Hi II hybrid line is a *c1 r1* double mutant; therefore, progenies (T_2_) from crosses of *c1*-*dHel-GFP* primary transformants(T_1_) with corresponding donor lines are all colorless kernels regardless of the potential existence of *aHels* in it (Figure S1). Our *c1* tester line contains a functional *R1-st* gene; when crossing with it, the transgenic copy of *c1*-*dHel-GFP* is feasibly discriminated from the endogenous *c1* gene by purple pigmentation phenotypes resulting from somatic excisions of *dHel-GFP* in the mutable *c1*-*dHel-GFP* catalyzed by *aHels*. 

We first cross the T_2_ transgenic lines to the *c1* tester line to observe the F_1_ seed phenotype. Then, we crossed the BC_1_ generation (F_1_ × *c1* tester) of all transgenic lines with the inbred lines listed in Table S2 to assess the possibility of the presence of autonomous *Helitrons* in these genetic backgrounds via an expected spotting phenotype segregating in the BC_1_F_1_ populations. 

*C1-I*, an allele of the *C1* locus specifying a colorless phenotype in the aleurone layer, suppresses the wild-type *C1* allele in the heterozygous condition [51]. Since *C1-I* does not affect the expression of the *C1* allele in heterozygous *C1/C1-I* seeds, it may negatively interfere with *C1* on a structural level [52]. Some of the inbred lines in Table S2 may have *C1-I* that are unlike candidates for detection of genetic activities of autonomous *Helitrons* in current experimental systems.

### 4.3. PCR Amplification of Somatic Excision Products and Circular dHel Intermediates

The *dHel-GFP* somatic excision products were examined by nested PCR on genomic DNA of the leaf blade from the third leaf of seedlings at the V2 stage with primer combinations of BF1/RB3, BF3/C1-14, and TF35S-1/C1R1 (Figure 1A, Table S3). Circular *dHels* were examined by nested PCR on the same set of genomic DNA samples using primer combinations of GFP-R3/GFP-R4, GFP-R1/GFP-4R, and 22ZEIN-1/GFP-R35S (color-coded arrows in Figure 7A, Table S3). All PCR amplifications were performed on a Bio-Rad CFX T100 instrument according to the 2 × Phanta Max Master Mix instructions (Vazyme, P525, Nanjing, China). 

### 4.4. Molecular Characterization of Variant Transcripts from Transgenes

Rapid amplification of cDNA ends (RACE) is an effective method to rapidly amplify the 5′ and 3′ ends of cDNA from low-abundance transcripts based on PCR. The total RNA was prepared from the same batch of maize seedling leaves with the Transzol reagent following the user manual (TransGen Biotech, Beijing, China). The DNA was degraded by DNase I (GenStar, Beijing, China) following the manufacturer’s protocol prior to RACE PCR. The 5′ and 3′ cDNA of the *c1* transgene was cloned by HiScript-TS 5′/3′ RACE Kit (Vazyme, RA101, Nanjing, China) following the manufacturer’s protocol.

## 5. Conclusions

Taking together, *c1-dHel-GFP* transgenic systems presented here are likely profitable in pursuing the genetic activity of autonomous *Helitrons* and elucidating the transposition mechanisms, RCR or cut-and-paste, of the transformed *dHels* catalyzed by the *aHels* at the molecular level. Meantime, complications of unexpected phenotypes of *c1-dHel-GFP* transgenic systems have been introduced serendipitously, and our research on the transcription alteration of the transgenes may aggrandize the functional characterization of the regulatory promoter regions, normally enriched with SNPs, indels or transposon insertions in plant. It is worthwhile to construct novel *c1-dHel-GFP* cassette in which *dHel* is inserted in the exons of the *c1* gene to lessen the intricacy of the entire system for an inspiring breakthrough in *Helitron* genetics.

## Figures and Tables

**Table 1:** Sequence features of *dHel-GFP* constructs and pigmentation phenotypes of T_2_ transgenic plants and F_1_ segregants of test cross population.

Construct	Site (nt)	*dHel-GFP* (bp)	Donor Line	T_1_ Events	T_1_ Single Copy	T_2_ Coleoptiles	T_2_ Anthers	F_1_ Kernel
S1-Hel1-4	−93	1723	B104	3	1	Purple	P	Mottled
S2-Hel1-4	−49	1723	B104	5	4	Purple	LP *	Mottled
S3-Hel1-4	−47	1723	B104	5	4	Purple	LP	Mottled
S4-Hel1-4	1	1723	B104	6	2	Colorless	Colorless	Colorless
S3-Hel1613	−47	1781	B104	5	4	Purple	LP	Colorless
S3-Hel1158	−47	1996	B104	5	4	Purple	LP	Mottled
S3-HelA2	−47	2038	HiⅡ	5	4	Purple	LP	Mottled

Note: Single copy T_2_ events were used for RACE. All T_2_ events and corresponding F_1_ were used for *Helitron* somatic excision footprint analysis. * LP, light purple.

**Table 2:** Somatic excision activity in F_1_ segregants from different *c1-dHel-GFP* T_2_ transgenic plants.

Constructs	Examined Segregants	Segregants with Detected SE	T-DNA Copy Numbers in Transformants
S1-Hel1-4	14	6	2 (Arrayed in tandem)
S2-Hel1-4	36	0	1
S3-Hel1-4	34	0	/
S4-Hel1-4	15	3	1
S3-Hel1613	34	0	/
S3-Hel1158	15	1	1
S3-HelA2	17	2	1

**Table 3:** Sequence of full and empty sites from *dHel-GFP* constructs.

*dHel-GFP* Construct	*dHel* 5′-End Junction *dHel* 3′-End Junction
S3-Hel1158	ACGTCGACCGCGCGCGTGCA**TCTATACTAT…AACCGACTAG**TTTAAATACGCCGACGACGG
S3-Hel1158 (e)	ACGTCGACCGCGCGCGTGCA---------------------------------------------TTTAAATACGCCGACGACGG
S3-HelA2	ACGTCGACCGCGCGCGTGCA**TCTCTACTAC…ACTCACCTAG**TTTAAATACGCCGACGACGG
S3-HelA2 (e)	ACGTCGACCGCGCGCGTGCA---------------------------------------------TTTAAATACGCCGACGACGG
S1-Hel1-4	GTTAGCGCCACTGCTACAGA**TCTATACTAC…ACCTAACTAG**TCGAGGCACCGGTCAGCCGG
S1-Hel1-4 (e)	GTTAGCGCCACTGCTACAGA---------------------------------------------TCGAGGCACCGGTCAGCCGG
S4-Hel1-4	CGACGAGAGAGCGAGCGCGA**TCTATACTAC…ACCTAACTAG**TGGGGAGGAGGGCGTGTTGC
S4-Hel1-4 (e)	CGACGAGAGAGCGAGCGCGA---------------------------------------------TGGGGAGGAGGGCGTGTTGC

Note: *Helitron* sequences are in bold; e: empty site.

## Data Availability

Not applicable.

## Supplementary Materials

The following supporting information can be downloaded at: https://www.mdpi.com/article/10.3390/ijms24076574/s1.
