# Supplementary material for: Abundance of Transgene Transcript Variants Associated with Somatically Active Transgenic Helitrons from Multiple T-DNA Integration Sites in Maize
Source: Int J Mol Sci. 2023 Mar 31;24(7):6574. doi: 10.3390/ijms24076574 (PMC10095026; doi:10.3390/ijms24076574)
Supplement: Supplementary file 1 [file ijms-24-06574-s001.zip › ijms-2299540-supplementary.pdf]

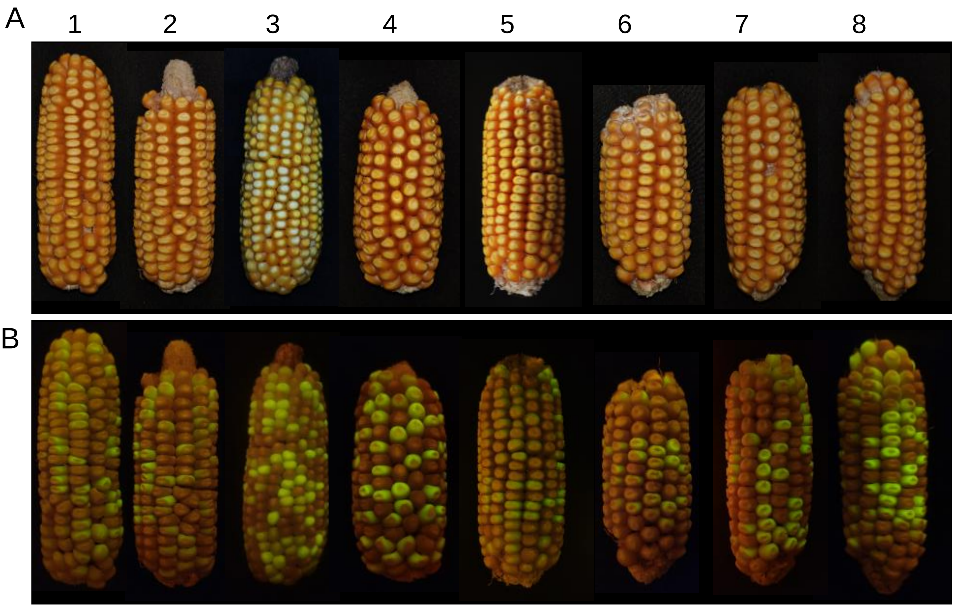

Figure S1. Kernel phenotype of T_2 ears. **A,** Representative T_2 ears from crosses of primary transformants with corresponding donor lines for various constructs. Anthocyanin pigmentation is invisible in all T_2 ears for primary transformants harboring individual *c1-dHel-GFP* constructs. **B,** GFP fluorescence, as detected under blue light illumination of the same T_2 ears as in (A), showing the segregation of fluorescent kernels. 1, S3-Hel1613; 2, S3-Hel1158; 3, S3-HelA2; 4, S1-Hel1-4; 5, S2-Hel1-4; 6, S3-Hel1-4; 7, S4-Hel1-4; 8, positive *C1* transgenic line.

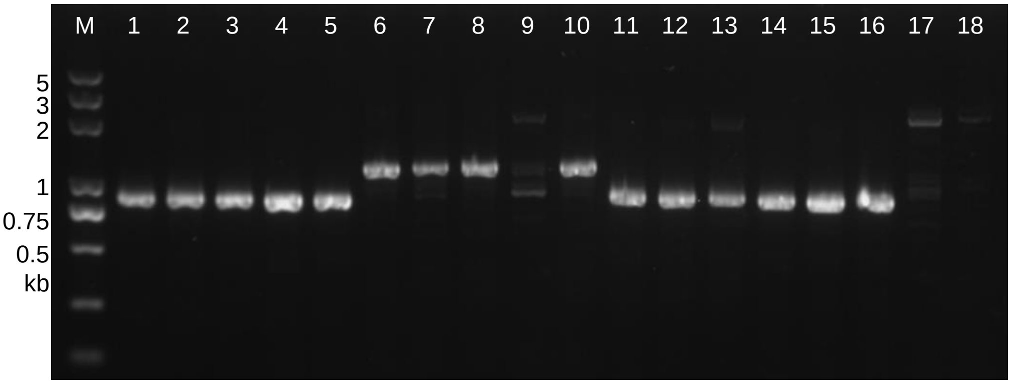

Figure S2. An RNA expression analysis of *C1* in leaf tissue of T_2 transgenic seedlings. RT-PCR primer pairs (C1-5F&C1-3R) are shown in Figure 1A and primer sequences are shown in Table S3. Lane 9 corresponding to one event of S4-Hel1-4 with transcriptional silencing. Transgenic events of Lane 1, 2, 4, 7, 11, 13 and 15 were used for 5′ and 3′ RACE. Lanes 1, S3-Hel1613; lanes 2-3, S3-Hel1158; lanes 4-5, S3-HelA2; lanes 6-10, S4-Hel1-4; lanes 11-12, S2-Hel1-4; lanes 13-14, S3-Hel1-4; lanes 15, S1-Hel1-4; lanes 16, positive *C1* transgenic line; lanes 17, B104 donor line; lanes 18, Hi II donor line.

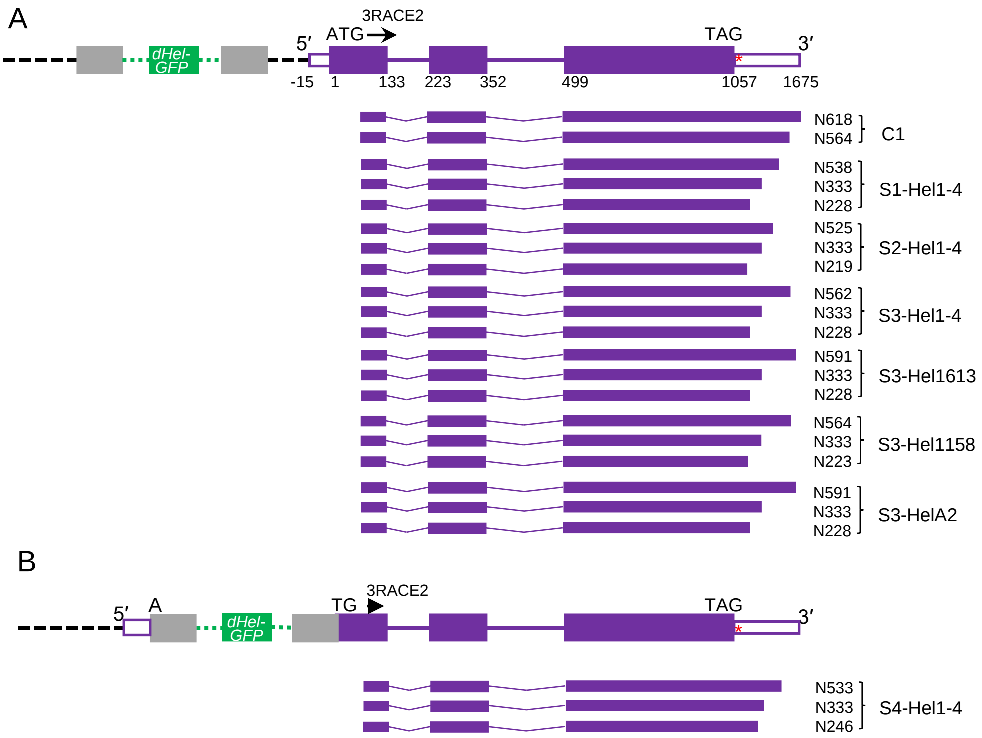

Figure S3. Schematic representation of *C1* transcript variants detected by 3′ RACE from T_2 transgenic leaf tissue. *GFP* promoter (azs22-10 promoter) and terminator (CAMV 35S terminator) are indicated by orange and red dotted lines respectively. *C1* exons and introns are indicated by purple boxes and lines respectively. *C1* promoter is indicated by black dotted line. 5′ and 3′ UTR are represented by open boxes. Alternative splicing events at non canonical sites are shown with broken lines. *dHels* are indicated by grey boxes and GFP is indicated by green boxes. Horizontal arrows, primer for 3′ RACE. **A,** *C1* transcripts detected in total RNA extracted from leaf tissue of T_2 transgenic plants harboring *dHel* insertions in the *C1* promoter region (sites S1 to S3); **B,** *C1* transcripts detected in total RNA extracted from leaf tissue of T_2 transgenic plants harboring the *dHel* insertion at the translation start site of the *C1* gene (site S4).

TAGACAACA*AGTACACG***TATAGATGTCCAATAAGCACGAGGCCCGCGAGCCCGGCACGAAGCC** 60

CGCTTTTTGGGCCCGGTCCGAGCCCGGCTCGGCCCGGTTATATGCAGACCCGGGCCGGCC 120

CGGCACGAATAAGCGGGCCGGGCTCGGACAGGAAATTAGGCACGGTGAGCTAGCCCGGCA 180

CGGCCCGTTTAGGTCTAAGCCCGTTAAGCCCGTTTTTTTACACTAAAACGTGCTTCTCGG 240

CCCGCATAGCCCGCTTCTCGGCCCGCTTTTTTCGTGCTAAACGGGCCGGCCCGGCCCGTT 300

TAGGCCCGTTGCGGGCCGGGCTCGGACAGGAAATTGAGCCCGCGTGCTTAGCCGTCCCGG 360

CCCGGTTTTTTAATCGTGCCTGGCGGGCCAGGCCCAAAACGGGCCGGGCTTCACCGGGCC 420

CGGGCCGGACCGGGCCGGGCGGCCCGTTTGGACATCTCTA*AGTACACG*TATGGAGGAGAA 480

TATATATATAGTCATGCGTACGTATAGATTTTTTCATCCGATCCCAACAGAAATACGTAT 540

GAAAATGCTCTTCGTTCTTTTTCATTTATCATATCTATACTATACTTAAAACACCAGTTT 600

CAACGGTCGTCATGCGTCATTTTTTTACAAATAACCCCTCACAGCTATTTCAAATTAATC 660

CGCTGCACGTCTATAGATGCCAAACGACGCCCAACACTAGCC 720

Figure S4. Schematic representation of 3‘-UTR polyadenylation sites in T_2 by 3‘ RACE. *C1* stop codon is indicated by red bold letters. The polyadenylation sites are highlighted in purple for wild-type *C1* transgenic lines and in green for *c1-dHel -GFP* transgenic lines, respectively (Table S4). The downstream sequence of the stop codon is numbered at the right side. The 446-bp *hAT* element is in bold letters, and the TSD sequences are in italic underlined letters.

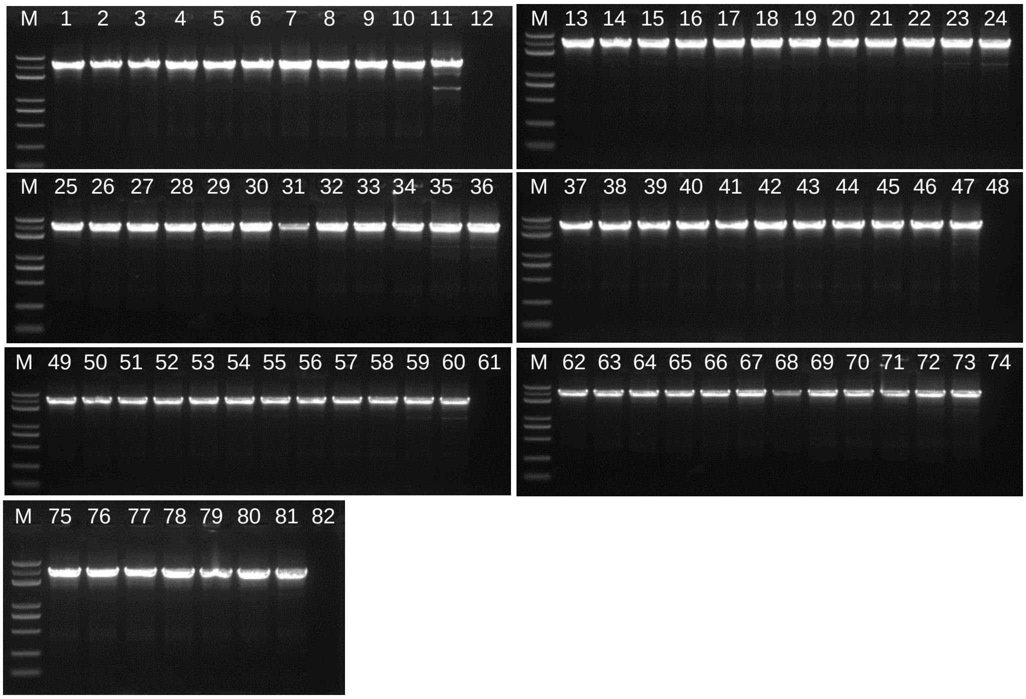

Figure S5. PCR amplification of somatic excision products for *dHel* in T_2 transgenic plants. Somatic excision products are detected via nest-PCR from some samples of T_2 transgenic plants. PCR primer pairs are shown in Figure 1A. Lanes 1–10, S3-Hel1613; lanes 13–22, S3-Hel1158; lanes 25–34, S3-HelA2; lanes 37–46, S3-Hel1-4; lanes 49–58: S4-Hel1-4; lanes 62–71, S2-Hel1-4; lanes 75–80, S1-Hel1-4; lanes 12, 48, 61, 74, 82: T_2 non-transgenic plants; lanes 11, 23, 24, 35, 36, 47, 59, 60, 72, 73, 81: F_1 somatic excision plants. DNA Ladder, from top to bottom, 5, 3, 2, 1, 0.75, 0.5, 0.25, 0.1 kb.

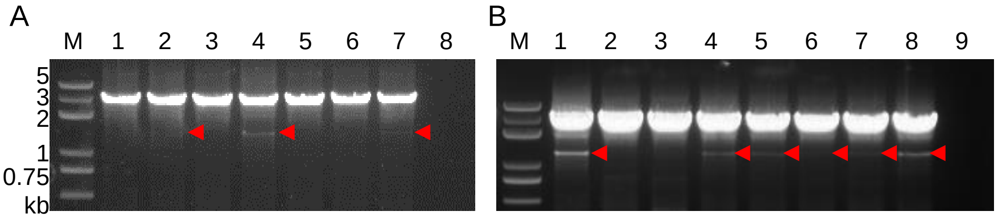

Figure S6. PCR amplification of somatic excision products for *dHel* in F_1 segregants from test-crosses between different *c1-dHel-GFP* T_2 transgenic plants and the *c1* tester line. PCR primer pairs are shown in Figure 1A. **A,** Lanes 1–7, F_1-*S4-Hel1-4*; lanes 8, F_1 individuals from a nontransgenic line crossed to the *c1* tester; **B,** lanes 1–8, F_1-*S1-Hel1-4*; lanes 9, F_1 individuals from a nontransgenic line crossed to the *c1* tester. Red arrows, PCR products with the expected size for *dHel* excision products.

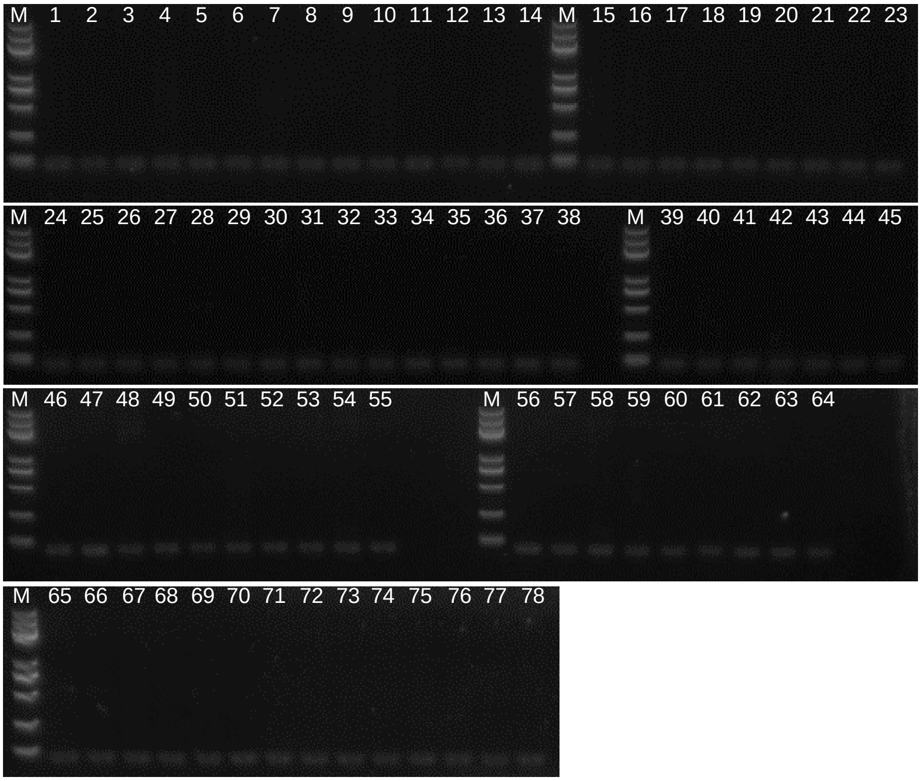

Figure S7. PCR detection of circular *dHel* intermediates from leaf tissues in F_1 segregants from test-crosses between different *c1-dHel-GFP* T_2 transgenic plants and the *c1* tester line. Lanes 1–14, S3-Hel1613; lanes 15–22, S1-Hel1-4, same DNA samples in lanes 1-8 from Figure S6B; Lanes 23, F_1 individuals from a cross between a non-transgenic line and the c1 tester; lanes 24–38, S2-Hel1-4, lanes 39–45, S4-Hel1-4, same DNA samples in lanes 1-7 from Figure S6A; lanes 46–55, S3-Hel1-4; lanes 56–64, S3-HelA2; lanes 65–78, S3-Hel1158. DNA Ladder, from top to bottom, 5, 3, 2, 1, 0.75, 0.5, 0.25, 0.1 kb.

Table S1. Sequence features of *dHel* transposons for reporter construction.

Helitron	Inbred line	Full length (bp)	Modified length (bp)	5' end (bp)	3' end (bp)
Hel1-4	H99	469	469	233	236
Hel1158	W22	1,158	739	430	309
Hel1613	B73	1,613	524	274	250
HelA2	B73	6,000	781	472	309

Table S2. Inbred lines in test-crossing for autonomous *Helitron* activities

Inbred line	Wild-type *C1*	Unilateral cross incompatibility (UCI)	Complete color inhibition (*C1-I*)
4722	Yes	No	No
A554	Yes	No	No
A619	No	No	No
A632	No	No	No
A679	No	No	No
B104	No	No	No
B37	No	No	No
B64	No	No	No
B68	No	No	No
B73	No	No	No
B76	No	No	No
C13	Yes	No	No
Chang7-2	Yes	No	No
CI66	No	No	No
CM 105	No	No	No
CM7	No	No	No
CML 10	No	No	No
CML 103	No	No	No
CML 108	No	No	No
CML 11	No	No	Yes
CML 14	No	No	No
CML 157Q	No	No	No
CML 158Q	No	No	No
CML 218	No	No	No
CML 220	No	Yes	No
CML 247	No	No	No
CML 277	Yes	No	No
CML 287	No	No	No
CML 314	No	No	No
CML 321	No	Yes	No
CML 322	No	No	No
CML 323	No	No	No
CML 328	No	No	Yes
CML 331	No	No	Yes
CML 45	No	No	No
CML 52	No	No	No
CML 91	No	No	No
CML 92	No	No	No
CML103	No	No	No

Table S2. *continued*

Inbred line	Wild-type *C1*	Unilateral cross incompatibility (UCI)	Complete color inhibition (*C1-I*)
H105W	No	No	No
H84	No	No	No
H99	No	No	No
HP301	No	Yes	No
HP72-11	Yes	Yes	No
IA5125	Yes	No	No
Ki11	No	No	No
Ki14	No	No	No
Ky228	Yes	No	No
M162W	No	No	No
Mo17	No	No	No
Mo24W	No	No	No
Ms71	No	No	No
N192	No	No	No
NC268	No	No	No
ND246	No	No	No
P39	Yes	No	No
Pa91	No	No	No
Qi319	No	No	No
Sg 1533	No	No	No
T232	No	No	No
Tzi 16	No	No	No
Tzi 16	No	No	No
Va102	Yes	No	No
Va26	No	No	No
Va35	Yes	No	No
W182BN	No	No	No
W64A	No	No	No
Wu-312	No	No	No
Zheng58	No	No	No
*c*1 tester	No	No	No

Table S3. Oligonucleotide Primers for PCR Analysis.

Experiments	Name	Sequence (5′ to 3′)
RT-PCR	C1-5F	CGAGAGAGCGAGCGCGA
	C1-3R	GTTGTCTACGCAAGCTGCC
3′-RACE	3RACE1	GGCGTGTTGCGCGAAGGAAGGCGTTAAG
	3RACE2	ACGATGCCTTGGCCGCCTACGTCAAGG
5′-RACE	5RACE1	GAAGAGTCCGCCCGTGCACCGCACG
	5RACE3	CGCTATTCTGGCCGGTCTCGCAGGC
Somatic excision	BF1	CGTGCTTGTCTCGATGTAGT
	BF3	GTTCTGGGCTCATGGTAGAT
	TF35S-1	TCCCTTACGTCAGTGGAGA
	RB3	CCAGGCTTTACACTTTATGCT
	C1-14	GATGAGCGGCGCTATTCTG
	C1R1	ACACGCATGTACATATGAATGAG
Circular *dHel-GFP*	GFP-R3	TGAACTTGTGGCCGTTTACG
	GFP-R4	GGTCCTGCTGGAGTTCGT
	GFP-R1	CCCGGTGAACAGCTCCT
	GFP-4R	ATCACTCTCGGCATGGACGA
	22ZEIN-1	CTACGTGGAATGACATGCA
	GFP-R35S	GTTCCTATAGGGTTTCGCTCAT

Table S4. Polyadenylation sites of *C1* transcripts by 3'-RACE from T_2 transgenic plants.

Constructs	Site 1	Site 2	Site3
S1-Hel1-4	228	333	538
S2-Hel1-4	219	333	525
S3-Hel1-4	228	333	562
S4-Hel1-4	246	333	533
S3-Hel1613	228	333	591
S3-Hel1158	223	333	564
S3-HelA2	228	333	591
Positive *C1*	564	618	/

Note: Polyadenylation sites, locations downstream of the *C1* stop codon.

Table S5. Sequence features of *C1* transcript detected by 5′ RACE.

Construct	Transcript in Figure 5	Sequence length (bp)	Initiation site
Positive *C1*	CK	472	Canonical -15 bp
S1-Hel1-4	A1	566	16 bp upstream from *dHel* 3'-end
	A2	472	Canonical -15 bp
	A3	561	Canonical -15 bp
S2-Hel1-4	A4	475	Canonical -15 bp
	A5	340	16 bp upstream from the 2nd exon
S3-Hel1-4	A6	660	67 bp upstream from *dHel* 3'-end
	A7	472	Canonical -15 bp
	A8	618	Canonical -15 bp
	A9	340	16 bp upstream from the 2nd exon
S3-Hel1613	A10	472	Canonical -15 bp
	A11	340	16 bp upstream from the 2nd exon
S3-Hel1158	A12	472	Canonical -15 bp
	A13	340	16 bp upstream from the 2nd exon
S3-HelA2	A14	2560	20 bp upstream from *dHel* 5'-end
	A15	971	20 bp upstream from *dHel* 5'-end
	A16	1018	19 bp upstream from *dHel* 5'-end
	A17	908	44 bp downstream of *dHel* 5'-end
	A18	970	30 bp downstream of *dHel* 5'-end
	A19	517	13 bp upstream from *dHel* 3'-end
	A20	458	1 bp upstream from 1st exon
	A21	455	nucleotide position 3 in the 1st exon
S4-Hel1-4	B1	2250	68 bp upstream from *dHel* 5'-TC
	B2	1917	67 bp upstream from *dHel* 5'-TC
	B3	793	18 bp upstream from *dHel* 5'-TC
	B4	559	12 bp upstream from *dHel* 5'-TC
	B5	523	67 bp upstream from *dHel* 3'-end
	B6	626	nucleotide position 67 in the 1st exon
	B7	544	nucleotide position 3 in the 1st exon
	B8	534	nucleotide position 70 in the 1st exon
	B9	388	nucleotide position 70 in the 1st exon
	B10	486	16 bp upstream from the 2nd exon
	B11	340	16 bp upstream from the 2nd exon

Note: Putative wild type *C1* transcripts are in red.

Table S6. Sequence patterns surrounding the transcription start site (TSS) of *C1* transcript detected by 5′ RACE.

Dinucleotides flanking TSS (±1 bp)	Transcript variants in Figure 5	TATA-box location to TSS	TATA-box motif	CG content between TATA-box and TSS (%)
CA	A1	-34 to -31	TATA	47
	A5	-36 to -28	ATATATATA	70
	A6	-35 to -29	TACAAAA	57
	A9	-36 to -28	ATATATATA	70
	A11	-36 to -28	ATATATATA	70
	A13	-36 to -28	ATATATATA	70
	A14	ND	ND	ND
	A15	ND	ND	ND
	A16	ND	ND	ND
	B1	ND	ND	ND
	B5	-35 to -29	TACAAAA	57
	B10	-36 to -28	ATATATATA	70
	B11	-36 to -28	ATATATATA	70
CG	CK (pTSS)	-33 to -26	ATTTAAATA	62
	A2	-33 to -26	ATTTAAATA	62
	A3	-33 to -26	ATTTAAATA	62
	A4	-33 to -26	ATTTAAATA	62
	A7	-33 to -26	ATTTAAATA	62
	A8	-33 to -26	ATTTAAATA	62
	A10	-33 to -26	ATTTAAATA	62
	A12	-33 to -26	ATTTAAATA	62
	A20	-46 to -39	TTTAAATA	68
	B6	ND	ND	ND
TG	A17	-31 to -28	TATA	52
	A21	-49 to -42	TTTAAATA	66
	B7	-51 to -48	TATA	49
	B8	ND	ND	ND
	B9	ND	ND	ND
AC	B2	ND	ND	ND
AG	A18	-17 to -14	TATA	50
	B4	-36 to -29	TTTAAATA	64
GA	B3	-30 to -23	TTTAAATA	68
GG	A19	-44 to -37	TTTATATA	36

Note: pTSS, primary transcription start site; ND, not detected

Table S7. Circular *dHel* intermediates detection in different populations.

Construct	Expected size (bp)	PCR products in T_2 (bp)	PCR products in F_1 (bp)
S1-Hel1-4	651	345	500
S2-Hel1-4	651	ND	ND
S3-Hel1-4	651	ND	ND
S4-Hel1-4	651	ND	ND
S3-Hel1613	706	ND	ND
S3-Hel1158	920	ND	593
S3-HelA2	963	ND	ND

Note: The expected circular *dHel* intermediates include both intact 5′-TC and 3′-CTAG sequences. F_1 segregants from a test-cross between different *c1-dHel-GFP* transgenic plants and the *c1* tester line. Sequence analysis showed that PCR amplifications from T_2-S1-Hel1-4 (Figure 7 B5), F_1-S1-Hel1-4 (Figure 7 C38), F_1-S1-Hel1158 (Figure 7 C9) are 345 bp, 500 bp and 593 bp in length, respectively (Figure 7D). ND, not detected.

Table S8. Palindromic sequences in *dHel-GFP* constructs.

dHel	Palindromic sequences	Location to 3′-end CTAG (bp)
Hel1-4	AAATCGAGATTT	33
	TTTTTACCAAAAA	210
Hel1613	CCCGTTGCAACGCACGGG	11
Hel1158	TAAAAAAATCTTGAAATTTTTTTA	58
HelA2	TTTATATATTTCATACAAA	39
	TTTACAAAATTCTAAA	141
